# Artificial Intelligence as an ally to human reproduction and embryology

**DOI:** 10.5935/1518-0557.20200065

**Published:** 2021

**Authors:** Luiz Mauro Oliveira Gomes, Camila Dutra de Souza Francisquini

**Affiliations:** 1 Reproferty, Center of the Human Reproduction, São José dos Campos, Brazil

John McCarthy coined the term Artificial Intelligence (AI) at the Dartmouth Summer Research Project on Artificial Intelligence in 1955. Artificial Intelligence is defined as the ability of machines to learn and display intelligence, and it has developed rapidly and gradually in our personal and social lives. In recent years, computers driven by energy, memory, storage and with large amounts of data, have been dealing with increasingly complex learning tasks with incredible success ^(Deo, 2015)^.

Machine Learning (ML) is based on the idea that we can build machines to process data and learn on their own, without our constant supervision. Machine Learning is a way to get Artificial Intelligence. ML algorithms use statistics to find patterns in large amounts of data. In short, AI and ML have an unbiased approach to multiparameter analyses.

The purpose of reproduction treatment is a healthy live birth, and despite advances in ovarian stimulation, extended embryo culture, genetic testing preimplantation and embryo selection, on average, only a third of all *in vitro* fertilization cycles result in pregnancy. Due to the growing need to create tools to help improve treatments without losing individualization, studies have started using Artificial Intelligence in Human Reproduction ^(Curchoe & Bormann, 2019)^.

In the context of assisted human reproduction, almost all aspects have been guidelines for scientific studies, for example, concerning the male factor, we have used it in sperm morphology and identification. Concerning the female factor, since the development of ideal protocols for controlled ovarian stimulation for *in vitro* fertilization, such as the prediction of empty follicles or with oocytes;in the scope of embryology, such as predicting the formation of blastocysts from oocytes, assessing and predicting the quality of human blastocysts, predicting live births by blastocyst and improving embryonic selection in order to reduce the number of cycles and achieve the goal of a healthy live birth.

Embryo selection is based on subjective analysis of development and morphological characteristics, and the quality or number of points required to classify an embryo as fit for transfer. It has been widely explored, for instance concerning the thickness of the zona pellucida, granularity cytoplasm, multinucleation, pronuclei singamia, number of blastomeres, degree of fragmentation, blastocel size, cleavage time and, more recently, morphokinetics.

The importance of embry selection and its development was the natural starting point for the application of AI in the *in vitro* fertilization laboratory, and due to the availability of high-quality image data and advances in time lapse technologies, it has been possible to develop embryo selection algorithms to achieve a successful *in vitro* fertilization cycle ^(Capalbo et al., 2014)^.

In 2008, the first Time Lapse System (TLS) on the market was sold for use in IVF (Primo Vision™, Vitrolife, Gothenburg, Sweden); and today we have the time lapse system integrated with the incubator, which are: EmbryoScope^®^ and EmbryoScopePlus (FDA approved, Vitrolife); Miri^®^ (ESCO, Egaa, Denmark); Geri^®^ (Genea Biomedx, Sydney, Australia). Numerous experimental methods have been developed to analyze implantation potential and live births rate of *in vitro* fertilization embryos from images or video, including morphokinetic analyses available through the Time Lapse System ^(Wong et al., 2010; Conaghan et al., 2013; Kirkegaard et al., 2012; Rubio et al., 2014)^.

Advances in Time Lapse System technologies have led to the development of embryo selection algorithms and the higher-powered computer processing to analyze a large set of images, combining parameters, which may be linked to embryonic viability.

The first predictive approach from time-lapse images came from developing an algorithm capable of automatically quantifying the duration of cytokinesis and the times between mitoses, up to the 4-cell stage ^(Wong et al., 2010)^. This system was marketed under the name of English Early Embryo Viability Assessment (Eeva™), being the first clinically validated platform that integrates time-lapse, two predictive parameters and automatic software.

^Khosravi et al. (2019)^ used AI and Time Lapse with a model they developed, capable of predicting the blastocyst quality with an AUC>0.98, after analyzing more than 10,000 embryos. Using a similar approach, ^Tran et al. (2019)^ recently reported the development of a deep learning model for automatically recording morphokinetic videos. The authors retrospectively analyzed more than 10,000 videos from various centers and were able to show that their model could identify images of blastocysts that yielded a fetal heartbeat, with an AUC> 0.90.

Two studies, presented at the American Society for Reproductive Medicine (2018), ^Zaninovic et al. (2018a)^, reported that AI enhanced the quality evaluation of blastocysts. The first entitled “Assessing human blastocyst quality using artificial intelligence (AI) convolutional neural network (CNN)”, using deep neural networks with Google's Inception architecture. They evaluated 50,392 images of 10,148 embryos grown in a Time Lapse system, and found 97.52% accuracy to classify good and bad blastocysts. With 90.6% accuracy for the true positive (Blastocyst implanted and of good quality) and 89.6% accuracy for the true negative (Blastocyst not implanted and of low quality).

The second study, with other research group, the ^Zaninovic et al. (2018b)^ presented “Application of artificial intelligence technology to increase the efficacy of embryo selection and prediction of live birth using human blastocysts cultured in a time-lapse incubator”, in which they evaluated 303 embryos from single blastocyst transfers that resulted in live births, using an artificial natural network architecture associated with agenetic algorithm, analyzing 386 time lapse images of embryos that remained in culture for 111.5h after ICSI. They reported that the precision in predicting live births based on morphokinetic data was 83% and the overall live birth prediction accuracy under AI using image analysis was 85%.

^Meseguer Escriva et al. (2018)^ presented at the 34^th^ ESHRE Annual Meeting, a study “Using artificial intelligence (AI) and time-lapse to improve human blastocyst morphology evaluation”. In this, the agreement was assessed using confusion matrices, ROC curves and Kappa Index, in 223 images of human embryo taken at 111.5h after ICSI, graded for Internal Cellular Mass (ICM), trophectoderm and expansion using Gardner grading systems. The AI’s overall accuracy for predicting blastocyst expansion is: training 93.9% and validation 81.5%; and factor prediction of ICM: training 93% and validation 78,8%; and trophectoderm: training 78,8% and validation 78,3%. The AI system was considerably more predictive of expansion (AUC 0,888-0,956) compared to ICM (AUC0,605-0,854) and trophectoderm (AUC 0,726-0,769).

In a survey evaluating retrospective data from previous IVF/ICSI records, the authors predicted the outcomes with an accuracy of more than 80%.They found that the woman's age, the number of embryos developed and serum estradiol level on the day of HCG administration were better predictive factor ^(Hafiz et al.,2017)^.

At the virtual ^ESHRE 36^th^ Annual Meeting (2020)^, some studies were published on Artificial Intelligence in Human Reproduction. ^Bori et al. (2020)^ used the KIDScoreD5 algorithm to identify embryos with normal chromosomal status and high potential for obtaining a live birth. This algorithm classifies embryos into categories based on the points of cleavage times and blastocyst appearance. It was a retrospective analysis including 22,461 embryos, and they found that embryos rated higher, had statistically higher implantation rates and live birth rates. This study showed the KIDScoreD5’s ability to distinguish between embryos with similar morphological characteristics, which would have the greatest potential, concluding that this algorithm can help the embryologist in decision making.

^VerMilyea et al. (2020)^ presented another study in which they used computer vision image annotation techniques to compose artificial intelligence, providing a reliable and robust assessment of blastocysts, with different types of cameras, microscopes and focal lengths. They concluded that in all these variables, the results suggest that this method of pre-processing and automatic annotation, as well as AI trained in a globally diverse data set, creates a generalizable AI that is robust to the type of camera and focal configuration, regardless of hardware.

AI has also been considered as a tool to pre-screen embryos and identify those with a low probability of being genetically altered, that way, only a few embryos would go to PGT-A, preventing all embryos from having to be biopsied and going for genetic analysis ^(Gleicher et al., 2018)^. Another aspect, is that AI can be used as a quality control tool in thawing embryos, or even, monitoring embryo culture systems throughout the year.

Based on all the possibilities for using Machine learning and Artificial Intelligence tools, and because it is a clearly growing methodology in human reproduction and embryology, it is well known that this technology can be part of the routine in human reproduction clinics. Taking into account that every step of preparing the algorithm, source code, data training and data validation are performed satisfactorily and obtain high accuracy and precision to benefit everyone involved.
